# Decrease in seroprevalence of Hepatitis A after the implementation of nationwide disposable tableware use in Taiwan

**DOI:** 10.1186/1471-2458-10-719

**Published:** 2010-11-23

**Authors:** Shih-Bin Su, Ching-Yih Lin, Ming-Jen Sheu, Wei-Chih Kan, Hsien-Yi Wang, How-Ran Guo

**Affiliations:** 1 Department of Family Medicine, Chi-Mei Medical Center, Tainan, Taiwan; 2 Institute of Biomedical Engineering, Southern Taiwan University, Tainan, Taiwan; 3 Department of Gastroenterology, Chi-Mei Medical Center, Tainan, Taiwan; 4 Department of Nephrology, Chi-Mei Medical Center, Tainan, Taiwan; 5 Department of Environmental and Occupational Health, College of Medicine, National Cheng Kung University, Tainan, Taiwan; 6 Department of Occupational and Environmental Medicine, National Cheng Kung University Hospital, Tainan, Taiwan; 7 Sustainable Environment Research Center, National Cheng Kung University, Tainan, Taiwan; 8 Center for Occupational and Environmental Health and Preventive Medicine, National Cheng Kung University, Tainan, Taiwan

## Abstract

**Background:**

Taiwan is an endemic area of viral hepatitis, including hepatitis A, which is transmitted mainly from the fecal-oral route. In order to reduce the transmission through food intake, the government implemented a policy of nationwide disposal tableware use in public eating places in 1982. We conducted a study to estimate the seroprevalence of Hepatitis A in a group of workers in Taiwan in 2005, determine the risk factors, and compare seroprevalence to published estimates in Taiwan to evaluate changes in the seroprevalence after the implementation of the nationwide disposal tableware use.

**Methods:**

We recruited workers of an industrial park during their annual health examinations in 2005 and measured their anti-hepatitis A virus IgG titer using microparticle enzyme immunoassay. We compared the seroprevalence across different birth cohorts within the study population and also analyzed data from previous studies.

**Results:**

The overall sero-positive rate was 22.0% in the 11,777 participants. The rate was much lower among those who were covered by the program since birth (born after 1982) in comparison with those who were not (2.7% vs. 25.3%, p < 0.001). From the analyses of data from pervious studies, we found the age-specific rates were similar in cohorts born in or after 1982 across studies conducted in different time periods but decreased with the calendar year in cohorts born before 1982. In particular, the age-specific seroprevalence dropped to less than one third in a three-year period among those who were born around 1982.

**Conclusions:**

Data from both the current and previous studies in different time periods supported the effectiveness of disposal tableware in preventing the transmission of hepatitis A.

## Background

Hepatitis A is an important global public health problem, and the pathogen (hepatitis A virus; HAV) is mainly transmitted through the fecal-oral route[1,2]. Therefore, hepatitis A is more prevalent in the under-developed and developing countries where environmental sanitation is poor and people have poor hygiene practices[1]. Hepatitis A is usually asymptomatic in children but may cause clinically apparent disease in adults. Its severity increases with age, and although most cases have complete recovery without sequelae, the mortality rate can reach 1.8% in patients over 50 years of age[1]. Residents in endemic areas often acquire the disease in the childhood, and the immunity after infection probably lasts for life[1,2]. Taiwan has been known as an endemic area of hepatitis A since the 1970 s, and the seroprevalence was more than 90% among adults in many surveys, with most HAV infections occurring in childhood[3-8].

Taiwan is also an endemic area of hepatitis B, and the high seroprevalence was documented even earlier[3,9]. Because hepatitis B may have severe sequelae, including liver cirrhosis and hepatocellular carcinoma, the Department of Health had implemented nationwide intervention programs to prevent and control hepatitis since the early 1980s[10]. The intervention programs included the use of disposable tableware in public eating places such as restaurants and food stands, which was enforced by laws and regulations in 1982. According to a government report[11], the public health programs or policies implemented in Taiwan from 1982 to 1986 included a regulation on the handling of garbage in the cities and a regulation on the construction of sewer in 1984, a regulation on the disposal of wastes in 1985, and a regulation on the hygiene of public eating places in 1986. No vaccination programs or polices were implemented during this period, but in 1980, the compulsory vaccination program for small pox was changed to a voluntary program, and the promotion for BCG vaccination was implemented. From 1982 to 1983, there were no other related health programs or policies noted.

Whereas the main target of disposable tableware program was to prevent hepatitis B, it was later realized that hepatitis B was mainly transmitted from the mother during delivery, and therefore disposable tableware has little role in its prevention[12]. On the other hand, because hepatitis A is mainly transmitted by the fecal-oral route, such a measure might be more effective in preventing hepatitis A. Therefore, we conducted a study to (1) estimate the seroprevalence of hepatitis A in an adult sample of industrial park workers in Taiwan in 2005, (2) determine which characteristics of the 2005 adult working cohort are associated with increased likelihood of being seropositive for hepatitis A, and (3) compare these estimates to published seroprevalence estimated in Taiwan in earlier years in order to evaluate changes in the seroprevalence of hepatitis A after the implementation of disposal tableware use.

## Methods

We analyzed the data of a hepatitis A serosurvey from workers of a company located in an industrial park in the Tainan area who received their annual health examinations at the clinic of the park in 2005. All the employees of the company (11,830 workers) are required to take a periodical health examination every year, which includes complete blood counts, hepatitis markers, and biochemistry tests of the blood sample. The participants were asked to fill out a standard questionnaire, which included questions on demographic data, history of hepatitis A, and vaccination for hepatitis A. Workers who received hepatitis A vaccination were excluded from further analyses.

Serum samples were obtained by clotting and centrifuging the blood at the room temperature and were then frozen and stored at -30°C. Anti-HAV IgG was determined by microparticle enzyme immunoassay (MEIA) (AxSYM HAVAB, Abbott Lab., North Chicago, IL, USA), and the assay has a cutoff index of 10 IU/l. All samples were analyzed within three days.

To evaluate impacts of the nationwide disposable tableware use, we compared the seroprevalence of hepatitis A among participants born at different time periods and paid particular attention to the changes before and after 1982. To evaluate impacts of the policy further, we used "hepatitis A" combined with "Taiwan" as key words to conduct a thorough search of literature on the seroprevalence of hepatitis A in Taiwan using PubMed. In addition, we conducted further searches through the references of the retrieved articles. We calculated and compared the seroprevalence rates of hepatitis A in different birth cohorts observed in different studies at different ages. Differences in prevalence rates were evaluated by chi-square or Fisher exact test at a two-tailed significant level of 0.05, and all the analyses were conducted using the SPSS (Version 15.0) software.

The protocol of this study was approved by the Research and Ethical Review Board of the Chi-Mei Medical Center. Because the analysis of the serosurvey data was done on the basis of existing medical records without referring to personal identification information, it was not required to obtain an informed consent from each individual.

## Results

All the 11,830 workers who received the annual health examination during the study period agreed to participate in the serosurvey, but we excluded 30 who had received hepatitis A vaccination before the study and 23 who did not complete the questionnaire. As a result, 11,777 participants (99.9%) were included in the data analysis. Their ages ranged from 19 to 57 years, but only 92 (0.8%) were 40 years of age or older (Table 1). Most participants (85.4%) were born before 1982, and there were more women than men (56.9% vs. 43.1%) (Table 1).

**Table 1:** Seroprevalence Rates of Hepatitis A among Different Subsets of the Study Population

Characteristics		Participants	Seropositive*	Rate (%)	p
All					
		11777	2592	22.0	
Gender					< 0.001
	Women	6704	1206	18.0	
	Men	5073	1386	27.3	
Age (year)					< 0.001
	15-19	162	3	1.9	
	20-24	2967	108	3.6	
	25-29	5461	959	17.6	
	30-34	2433	1050	43.2	
	35-39	662	405	61.2	
	≥40	92	67	72.8	
Year of birth					< 0.001
	≤1965	114	84	80.0	
	1966	72	48	66.7	
	1967	98	62	63.3	
	1968	119	84	70.6	
	1969	220	122	55.5	
	1970	247	139	56.3	
	1971	347	191	55.0	
	1972	391	181	46.3	
	1973	517	222	42.9	
	1974	621	238	38.3	
	1975	796	287	36.1	
	1976	1043	270	25.9	
	1977	1095	201	18.4	
	1978	1159	167	14.4	
	1979	1205	144	12.0	
	1980	1063	73	6.9	
	1981	951	32	3.4	
	1982	694	14	2.0	
	1983	413	12	2.9	
	1984	330	12	3.6	
	1985	191	9	4.7	
	1986	91	0	0.0	
Birth in or after 1982					< 0.001
	Yes	1719	47	2.7	
	No	10058	2545	25.3	
Childhood residence area					< 0.001
	Urban	4795	826	17.2	
	Rural	6216	1616	26.0	

*seropositive for IgG-anti-hepatitis A

With 2592 of the participants having positive tests, the overall seroprevalence of hepatitis A was 22.0%. The prevalence was higher in men (27.3% vs. 18.0%, p < 0.001) and generally increased with age (decreased with the calendar year of birth) (p < 0.001) (Table 1). In particular, the prevalence dropped rapidly from 12.0% to 3.4% between the 1979 birth cohort and the 1981 birth cohort, and those who were born in or after 1982 had a much lower prevalence than those who were born earlier (2.7% vs. 25.3%, p < 0.001) (Table 1). Participants who lived in rural areas during childhood had a higher prevalence than those who lived in the urban area (26.0% vs. 17.2%, p < 0.001). Furthermore, we found among the participants born before 1982, men had a higher prevalence (28.0% vs. 22.3%, p < 0.001), but among the participants born in or after 1982, men did not have a higher prevalence (0.8% vs. 2.9%, p = 0.252). Likewise, we found among the participants born before 1982, those who lived in rural areas during childhood had a higher prevalence (30.1% vs. 19.5%, p < 0.001), but among the participants born in or after 1982, those who lived in rural areas during childhood did not have a higher prevalence (3.2% vs. 2.5%, p = 0.448).

From the review of literature, we found three studies reporting age-specific seroprevalence rates of anti-HAV IgG in the Tainan area[7,8,12], six in the Taipei area[3,4,6,12-14], and one covering the whole southern Taiwan[15]. All the three studies in the Tainan area found that the seroprevalence generally increased with age, which was similar to the trends observed in the current study [6-8] (Table 2). In terms of birth cohorts, the prevalence rates dropped rapidly starting from the cohorts who were born around 1982 as observed in the current study. Specifically, it dropped from 26.1% in the 1974-1978 birth cohort to 4.9% in the 1979-1982 cohort in the study by Wu et al. [12], from 60.7% in the 1973-1976 cohort to 4.1% in the 1980-1981 cohort in the study by Liu et al. [7], and from 12.1% in the 1974-1978 birth cohort to 4.9% in the 1979-1982 cohort in the study by Wang et al. [8] On the other hand, the prevalence maintained below 3.1% after the 1981-1984 cohort in the study by Wu et al. [6], below 3.7% after the 1982-1983 cohort in the study by Liu et al. [7], and below 2.0% after the 1983-1985 cohort in the study by Wang et al. [8]. As in the current study, in all three previous studies, prevalence rates in cohorts born in or after 1982 were much lower than those in cohorts born before 1982 and were all below 5%. Furthermore, the age-specific rates were similar in cohorts born in or after 1982 across studies in different time periods but increased with the calendar year in cohorts born before 1982 (Table 2). None of the previous studies reported a prevalence rate for participants born in any single year, but we were able to compare the prevalence of cohorts born in certain periods covering a few calendar years between previous studies and the current study. The adults in the Liu et al.[7] and Wang et al.[8] studies were from the general population in the same area where we conducted the current study (Table 3), and the patterns of sero-prevalence were compatible with our observation. For people born in or after 1982, the prevalence rates in cohorts born in the same periods were not significantly different between the previous studies and the current study (p > 0.05 for all comparisons, except for the comparison of those who were born during 1983-1985 between the study by Wu et al. [12] and the current study), even though the proportions of members born in individual years were likely to be different across studies (Table 4). This indicated that the incidence of hepatitis A was very low from 1989, the year in which Wu et al. [6] began to draw samples, to the time when the current study was conducted.

**Table 2:** Comparison of Hepatitis A Seroprevalence Rate by Age in the Tainan Area in Different Studies

Study	**Wu et al., 1993**[12]			**Liu et al., 1994**[7]			**Wang et al., 2001**[8]			Current study		
(Time)	(1989-1991)			(1992)			(1998)			(2005)		
				
Age (years)	Year of birth	Case/Total	Rate (%)	Year of birth	Case/Total	Rate (%)	Year of birth	Case/Total	Rate (%)	Year of birth	Case/Total	Rate (%)
1-2	1987-1990	0/30	0.0	1990-1991	3/83	3.6	1996-1997	0/32	0.0			
3-4	1985-1988	1/32	3.1	1988-1989	2/70	2.9	1994-1995	0/59	0.0			
5-6	1983-1986	0/144	0.0	1986-1987	2/86	2.3	1992-1993	1/91	1.1			
7-8	1981-1984	5/268	1.9	1984-1985	3/82	3.7	1990-1991	1/124	0.8			
9-10	1979-1982	14/287	4.9	1982-1983	3/90	3.3	1988-1989	2/147	1.4			
11-12	1977-1980	19/315	6.0	1980-1981	3/73	4.1	1986-1987	0/74	0.0			
13-15	1974-1978	106/406	26.1	1977-1979	8/35	22.9	1983-1985	1/51	2.0			
16-19				1973-1976	17/28	60.7	1979-1982	2/41	4.9			
20-24				1968-1972	22/35	62.9	1974-1978	16/132	12.1	1981-1985	79/2580	3.1
25-29				1963-1967	26/37	70.2	1969-1973	16/44	36.4	1976-1980	855/5587	15.3
30-39				1953-1962	24/26	92.3	1959-1968	29/44	65.9	1966-1975	1574/3458	45.2
40-49				1943-1952	20/20	100.0	1949-1958	41/46	89.1	1956-1965	79/107	73.8
> 50				< 1942	25/25	100.0	< 1948	32/34	94.1	< 1955	5/8	62.5

**Table 3:** The three previous studies used for comparisons

Study	Study population	Test methods
Wu et al., 1993[12]	(1) 481 participants, aged 1-17 years, from a southern Taiwan village. (2) 1100 students randomly selected from 24251 elementary and junior middle school students in Tainan City.	EIA (HAVAB-EIA, Abbott Laboratories, North Chicago, IL)
Liu et al., 1994[7]	(1) 164 children under 3 years of age brought to the well-baby clinic. (2) 87 preschool children age 3-6 years from 3 kindergartens. (3) 311 children aged 6-16 years from two primary schools and one junior high school. (4) 176 adults, ranging from 16 to 67 years of age, including 34 pregnant women, recruited during health examination or prenatal care in the hospital.	RIA (HAVAB, Abbott Laboratories, North Chicago, IL)
Wang et al., 2001[8]	(1) 61 children under aged from well-baby clinic at the hospital. (2) 150 preschool children aged 3-6 years from three kindergartens. (3) 345 children, aged 6-12 years, from two primary schools. (4) 92 students selected at random from a junior high school. (5) 341 adults, 20-63 years of age, enrolled from health examination or prenatal care at the National Cheng Kung University Hospital.	RIA (HAVAB, Abbott Laboratories, North Chicago, IL)

**Table 4:** Comparison of Hepatitis A Seroprevalence Rates by Birth Years in the Tainan Area in Different Studies

Study (year)	**Wu et al., 1993**[12] (1989-1991)		Current (2005)			**Liu et al., 1994**[7] (1992)		Current (2005)			**Wang et al., 2001**[8] (1998)		Current (2005)	
Birth Years	Case/Total	Rate (%)	Case/Total	Rate (%)	Birth Years	Case/Total	Rate (%)	Case/Total	Rate (%)	Birth Years	Case/Total	Rate (%)	Case/Total	Rate (%)
1984-1986	0/17	0.0	21/612	3.4	1984-1985	3/82	3.7	21/521	4.0	1983-1985	1/51	2.0	33/934	3.5
1983-1985	0/127	0.0	33/934	3.5*	1982-1983	3/90	3.3	26/1107	2.3	1979-1982	2/41	4.9	163/3913	4.2
1982-1984	2/132	1.5	38/1437	2.6	1980-1981	3/73	4.1	105/2014	5.2					
1981-1983	3/136	2.2	58/2058	2.8										
1980-1982	5/145	3.4	119/2078	5.7										
1979-1981	9/142	6.3	249/3219	7.7										

*p < 0.05 for the Fisher's exact test for the difference in the prevalence rate between the previous study and the current study

**Figure 1:**
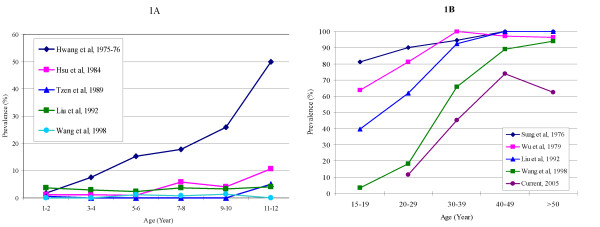
**Seroprevalence Observed in Different Age Groups in Different Studies**.

Three of the studies in Taipei were conducted before 1982[3,4,16]. Whereas the categorization of age varied across studies, the seroprevalence generally increased with age in all studies. In addition, more than 10% of the participants became positive by the age of 5, about half became positive by the age of 13, and more than 80% became positive by the age of 20 (Table 5 and Figure 1). The three studies after 1982 applied the same categorization of age and observed similar age-specific prevalence rates except for those who were born before 1982, even though the proportions of members born in individual years were likely to be different across studies[12-14] (Table 6). This indicated that the incidence of hepatitis A was very low from 1984, the year in which Hsu et al. [17] began to draw samples, to 1999 when Tseng et al. [14] began to draw samples. Furthermore, the prevalence rates observed among those who were born in or after 1982 were very low (not exceeding 3.2%) in all studies even up to the age of 12, indicating the incidence was low since 1982, as in studies in the Tainan area (Figure 1).

**Table 5:** Comparison of Hepatitis A Seroprevalence Rate by Age in the Taipei Area in Different Studies before 1982

Age	**Hwang et al. (1975-1976) **[16]			Age	**Sung et al. (1976) **[3]			Age	**Wu et al. (1979) **[6]		
					
(years)	Year of birth	Case/Total	Rate (%)	(years)	Year of birth	Case/Total	Rate (%)	(years)	Year of birth	Case/Total	Rate (%)
1	1974-1975	5/302	1.6	5-9	1967-1971	2/15	13.3	1-3	1976-1978	13/115	11.3
2	1973-1974	2/121	1.7	10-14	1962-1966	17/22	77.3	4-6	1973-1975	21/144	14.6
3	1972-1973	1/32	3.1	15-19	1957-1961	13/16	81.3	7-9	1970-1972	34/162	21.0
4	1971-1972	4/34	11.8	20-29	1947-1956	18/20	90.0	10-12	1967-1969	57/132	43.2
5	1970-1971	5/34	14.7	30-39	1937-1946	17/18	94.4	13-15	1964-1966	81/131	61.8
6	1969-1970	5/32	15.6	40-49	1927-1936	14/14	100.0	16-18	1961-1963	46/72	63.8
7-8	1967-1969	5/28	17.9	50+	1926-	12/12	100.0	19-24	1955-1960	126/155	81.3
9-10	1965-1967	7/27	25.9					25-29	1950-1954	8/8	100.0
11-12	1963-1965	13/26	50.0					30-39	1940-1949	31/31	100.0
13-14	1961-1963	37/43	86.0					40-49	1930-1939	64/66	97.0
15-19	1956-1961	46/50	92.0					50-78	1901-1929	134/139	96.4

**Table 6:** Comparison of Hepatitis A Seroprevalence Rate by Age in the Taipei Area in Different Studies after 1982

Age	**Hsu et al. (1984) **[17]			**Tzen et al. (1989) **[13]			**Tseng et al. (1999) **[14]		
			
(years)	Year of birth	Case/Total	Rate (%)	Year of birth	Case/Total	Rate (%)	Year of birth	Case/Total	Rate (%)
1-2	1982-1983	1/84	1.2	1987-1988	1/184	0.5	1997-1998	3/206	1.5
3-4	1980-1981	2/189	1.1	1985-1986	0/179	0	1995-1996	1/92	1.1
5-6	1978-1979	2/226	0.9	1983-1984	0/234	0	1993-1994	2/123	1.6
7-8	1976-1977	12/206	5.8	1981-1982	0/163	0	1991-1992	1/55	1.8
9-10	1974-1975	6/146	4.1	1979-1980	0/134	0	1989-1990	0/0	--
11-12	1972-1973	20/187	10.7	1977-1978	8/160	5.0	1987-1988	2/62	3.2
13-14	1970-1971	14/103	13.6						

## Discussion and conclusions

In this study we found the Taiwanese who were born after the implementation of nationwide disposable tableware use had a much lower seroprevalence rate of hepatitis A than that among those who were born before the implementation. In fact, even when participants who were born in 1981 were included, the current study observed a much lower prevalence rate in the age group 20 to 24 (born between 1981 and 1985) than that in 1998 in the study by Wang et al. [8] (participants born between 1974 and 1978; 79/2580 vs. 16/132, p < 0.001), and further lower than that in 1992 in the study by Liu et al. [7] (participants born between 1968 and 1972; 79/2580 vs. 22/35, p < 0.001) (Table 2). Before the implementation, a prevalence rate up to 81.3% in 1979 was observed by Wu et al. [6] in a similar age group (19 to 24 years old) in Taipei area (Table 4), and a prevalence rate up to 98% in the age group 20 to 29 was found in blood samples collected between November 1981 and February 1982 in the study covering the whole southern Taiwan [15]. The Tainan area is in the southern part of Taiwan, and the Taipei area is in the northern part of Taiwan. In the literature, we found one study in the central part of Taiwan, which observed a similar pattern of seroprevalence in blood samples collected from 2047 kindergarten children and 104 kindergarten teachers between 1995 and 1996 [18]. The prevalence in the children, who were 3 to 6 years old (born between 1989 and 1993) was 0.4%, while the prevalence in teachers, who were at least 18 years of age (born in or before 1978), was 78.8%. The population of an industrial park is not likely to be representative of the Taiwan population, especially with respect to demographic patters. However, in the current study and previous studies in Tainan and Taipei areas after 1982, the patterns of seroprevalence were very similar: those who were born after 1982 had similar prevalence at various ages, but the seroprevalence rates were different across different age groups in adults with a trend of increasing with age. In addition, the adults in the Liu et al.[7] and Wang et al.[8] studies were from the general population in the same area where we conducted the current study (Table 3), and the patterns of sero-prevalence were compatible with our observation. Furthermore, the sharp drop of seroprevalence around 1982 was observed across all previous studies. Therefore, it is reasonable to conclude that the impacts of disposable tableware use on the seroprevalence of hepatitis A observed in our study can be generalized to the whole country.

Although we found that being born in or after 1982 is a significant predictor for lower HAV seroprevalence, since the prevalence increases with age, using any cutoff year before 1982 may show the same results. Therefore, we conducted a post-hoc analysis comparing the prevalence among those participants in the current study who have lived almost all their lives in the disposable tableware era (born between 1981 and 1985 and thus were 20 to 24 years of age at the time of study) to that among those participants in the previous studies who were at the same age in the pre-disposable tableware era, using the data in Table 2. Because infants hardly use disposable tableware before 1 year old, such comparisons should be valid. Accordingly, we found the seroprevalence in this group of participants (3.1%) was significantly lower than that in those who were born between 1968 and 1972 in the Liu et al. study[7] (62.9%, p < 0.001) and that in those who were born between 1974 and 1978 in the Wang et al. study[8] (12.1%, p < 0.001). The seropositives rate decreased with age for those born after 1982 (Table 1), except those who were born in 1986. A possible reason for not seeing a seropositive case in those who were born in 1986 is that the number of participants (91) was too small to generate a stable estimate of the prevalence. As we can see from both Tables 1 and 2, most age groups without cases had less than 100 participants in the group.

Because HAV is mainly transmitted through the fecal-oral route, the use of disposable tableware can be an effective measure to reduce its transmission[2]. Nonetheless, other factors such as improvement in environmental sanitation might also contribute to the reduction in the seroprevalence of hepatitis A. However, there was no drastic improvement in environmental sanitation in Taiwan around 1982[11], and therefore the impacts of environmental sanitation were not likely to introduce the abrupt changes in the prevalence of hepatitis A as observed in the current study and previous studies conducted after 1982. Although a regulation on the construction of sewer was implemented in 1984 and might affect the seroprevalence of hepatitis A, it took a certain period of time for constructing the sewer system, and the seroprevalence had begun to decrease sharply several years before the implementation of this regulation. Likewise, the regulation on the handling of garbage in the cities implemented in 1984, the regulation on the disposal of wastes implemented in 1985, and the regulation on the hygiene of public eating places implemented in 1986 are not likely to account for the sharp decrease in seroprevalence starting from around 1982. Although a promotion for BCG vaccination against tuberculosis was implemented in 1980, it is not likely to affect the seroprevalence of hepatitis A. Only 30 participants (0.25%) in our study received HAV vaccination, and the vaccine was in fact not commercially available in Taiwan until 1995. Furthermore, currently the Taiwanese government has not recommended it for routine vaccination, and the high cost discourages people from receiving it on a voluntary basis. Therefore, even though the effectiveness of vaccination was close to 100%, the vaccinated rate is still very low in Taiwan. As a result, its impacts on the seroprevalence rates observed in the current study or other studies were very small. At the same time when the use of disposable tableware was implemented, the government also recommended using shared chopsticks and spoons, instead of using each individual's personal chopsticks and spoon, to take food and soup from shared containers. However, this preventative measure was not mandated by law, and therefore the compliance was not as good as the disposable tableware use. The compliance of disposal tableware use was reported to be near 99% in 1986[10]. When the government implemented the nationwide disposable tableware use in public eating places, the purpose was to reduce the occurrence of hepatitis, but not specifically for hepatitis A.

The three studies in the Taipei area and the study in central Taiwan before 1982 showed that most people contracted hepatitis A in childhood (more than 50% by the age of 15 years old) and that the seroprevalence reached 90% before 20 years of age[3,6,16,18]. Among our participants who were born in 1967 (15 years of age in 1982), the prevalence was 63.3% (Table 1). Since the incidence of hepatitis A was low after 1982, it is reasonable to infer that the prevalence of these participants at the age of 15 years old (in 1982) was over 50%, and therefore most members of our study population who were seropositive also acquired the disease in childhood. In Taiwan, rural areas generally have worse sanitation conditions than the urban areas, and several studies have shown that children living in rural areas had higher prevalence of hepatitis A than children living in urban areas. Therefore, it is reasonable to see the seroprevalence being higher in participants who lived in rural areas during their childhood when hepatitis A was prevalent[12,18,19]. Nonetheless, it should be noted that the difference was only significant among participants born before 1982, who made up 85.4% of the subjects, not among the participants born in or after 1982. This might be due to the small case numbers, because the relative risks were similar (1.5 vs. 1.3), or a reduction in the gap of general hygiene conditions between rural and urban areas over the years.

None of the three previous studies in the Tainan area assessed gender-specific seroprevalence rates of hepatitis A at different ages. One of the studies conducted in the Taipei area compared gender-specific rates at different ages but did not find any significant difference between the two genders[6]. However, the study was conducted before 1982, and the seroprevalence in the participants with ages similar to the participants born before 1982 in our study (> 23 years old) was nearly 100% in both men and women. A study conducted in the Taichung area after 1982 compared gender-specific rates in participants above 18 years of age (similar to our study) and observed a higher rate in men (100.0% vs. 76.8%)[18], which is similar to the findings in our study.

In addition to acquiring the disease and vaccination, immunity to HAV infection can also be obtained from maternal antibodies. The study conducted by Hwang et al. [16], which collected blood samples during 1975 and 1976 in Taipei, found 96.7% of the cord blood samples and 35.9% of samples obtained from infants between 1 to 11 months of age had HAV antibodies. The study by Wu et al. [6], which collected blood samples in 1979 in Taipei, also found that 100.0% of the cord blood samples and 32.1% of samples obtained from infants under 12 months of age had antibodies. Likewise, the three studies in Tainan area found seroprevalence ranging from 20.7% to 64.6% in infants[7,8,12]. However, the immunity decreased with age[6], and none of the seropositive infants in the study by Hwang et al. still had a detectable level of antibodies at follow-up, reflecting the loss of acquired maternal antibodies. In addition, the seroprevalence among 1-year-old children was only 1.6% in the study by Hwang et al., indicating that most of the acquired maternal antibodies were lost by the age of 2 years. Similarly, the study by Wu et al. [12] in the Tainan area found the congenital immunity decreased with age, from 75% among infants under 6 months of age to 11.8% among infants over 6 months of age, and all the three studies in the Tainan area found low seroprevalence in children 1 to 2 years old[7,8,12]. Because a substantial number of infants had acquired maternal antibodies against HAV, the participants in our study who were born in 1981 had similar prevalence rates to those who were born after 1982 (74/1719 vs. 32/951, p = 0.256), although the first year of their life span was not covered by the implementation of disposable tableware use. Other studies in Taiwan also observed similar prevalence rates between the two groups who were born around 1982 (Tables 2, 3, and 5).

With reduction in seroprevalence of hepatitis A in the population after the implementation of nationwide disposable tableware, the prevalence of infants carrying maternal antibodies also reduced in Taiwan. In the Tainan area, the seroprevalence rate in infants was 64.6% when Liu et al. [7] collected blood samples in 1992, but reduced to 20.7% when Wang et al. [8] collected samples in 1998 at the same hospital (p < 0.001). It can be expected that a remarkable reduction in the prevalence will be observed among infants given birth by mothers who were born after 1981. Furthermore, whereas hepatitis A is usually asymptomatic in children, it can cause clinically apparent disease in adults, and the severity increases with age. The 1982 birth cohort had became adults three years ago, and the number of adults susceptible to hepatitis A is increasing in Taiwan. The most commonly used disposable tableware in Taiwan includes plastic spoons, forks, bowls, dishes and cups, which had become a problem in terms of environmental protection. Therefore, since March 2006 the government has banned the use of disposable tableware in public eating places step by step starting from cafeterias and restaurants within governmental agencies. The findings from the current study are supportive of a protective effect of disposable tableware, in conjunction with additional sanitation and public health activities, in reducing HAV transmission in Taiwan. Even with improvements in water supply, food treatment, tableware cleaning, and environmental sanitation, it is uncertain whether hepatitis A will be re-emerging in Taiwan with the ban of disposable tableware. Therefore, nationwide campaign of improvement in personal hygiene and vaccination for people at high risk is desirable to offset the possible impacts of banning disposable tableware.

## Competing interests

1. Authors' declaration of personal interests: None.

2. Declaration of funding interests: This study was funded in full by Chi-Mei Medical Center (Grant No. CMFHR 9460).

## Authors' contributions

SBS conceived and designed the study, performed the statistical analyses, and was in charge of the recruitment of study participants. CYL, HYW, WCK and MJS helped the design of the study, collection of information, and interpretation of data. HRG participated in the study design, supervised the conduct of the study, and helped to draft the manuscript. All authors read and approved the final manuscript.

## Pre-publication history

The pre-publication history for this paper can be accessed here:

http://www.biomedcentral.com/1471-2458/10/719/prepub
